# Combining a CDK4/6 Inhibitor With Pemetrexed Inhibits Cell Proliferation and Metastasis in Human Lung Adenocarcinoma

**DOI:** 10.3389/fonc.2022.880153

**Published:** 2022-05-24

**Authors:** Yuan Ke, Cheng-Gong Liao, Zheng-Qing Zhao, Xiao-Min Li, Rong-Jie Lin, Long Yang, He-Long Zhang, Ling-Min Kong

**Affiliations:** ^1^Department of Oncology, Second Affiliated Hospital of Air Force Military Medical University, Xi’an, China; ^2^Department of Neurology, Changzheng Hospital, Naval Medical University, Shanghai, China; ^3^Department of Cell Biology, National Translational Science Center for Molecular Medicine, Air Force Military Medical University, Xi’an, China; ^4^State Key Laboratory of Cancer Biology, Xijing Hospital of Digestive Diseases, Air Force Military Medical University, Xi’an, China; ^5^Department of Hepatobiliary Surgery, Xijing Hospital, Air Force Military Medical University, Xi’an, China

**Keywords:** CDK4/6 inhibitor, pemetrexed, cell cycle, synergy, lung adenocarcinoma

## Abstract

**Background:**

Recent clinical trials of cyclin-dependent kinase 4/6 inhibitors (CDK4/6i) in human lung adenocarcinoma (LUAD) have not achieved satisfactory results. The disappointing results of single-drug treatments have prompted studies about synergistic therapies of CDK4/6i with other drugs. We aimed to test the anti-tumor effect of ribociclib (a CDK4/6i) combined with pemetrexed on LUAD and the potential mechanisms.

**Methods:**

Cell lines were exposed to ribociclib and pemetrexed at different doses. Antitumor effects were measured using growth inhibition. Cell cycle distribution and apoptosis were evaluated using flow cytometry. Cell migration and invasion were measured using wound healing and transwell invasion assays, respectively. The expression levels of proteins were analyzed using western blotting. Mice xenograft models were used for validation *in vivo*.

**Results:**

Synergism was associated with a combination of cell cycle effects from both agents. Cell cycle analysis revealed that pemetrexed blocked cells in the S phase, whereas ribociclib arrested cells in the G1 phase. Concomitant treatment with pemetrexed and ribociclib resulted in a significantly stronger antitumor ability than treatment alone. We also found that ribociclib strongly enhanced the pro-apoptotic activity of pemetrexed *via* the caspase/bcl-2 signaling pathway. In addition, we report for the first time that combination treatment with ribociclib and pemetrexed significantly inhibits the migration and invasion of LUAD cells.

**Conclusions:**

Combining ribociclib and pemetrexed showed a powerful ability to inhibit cancer proliferation, invasion, and metastasis, and it holds potential as a novel effective combinative therapy for patients with LUAD.

## Background

An increasing number of patients with lung cancer are being diagnosed with lung adenocarcinoma (LUAD) (1). For advanced LUAD without sensitizing mutations, such as EGFR, BRAF, ALK or ROS-1 gene rearrangements, chemotherapy with a platinum-based reagent is the main treatment option if no contraindication exists (2). Unfortunately, not all patients respond to first-line therapy, and even the patients who initially respond are likely to relapse (3). Treatment options for these patients remain an area of significant unmet medical need.

The cell cycle is regulated by multiple evolutionarily conserved process that is required for mammalian cell viability and progression (4). The uncontrolled cell cycle is a common feature of cancer. Cancer cells display unscheduled proliferation and genomic instability (5, 6). Targeting the cell cycle in cancer has been shown to be potential and promising therapeutic strategy (7). Cyclin-dependent kinase 4 (CDK4) and the closely related CDK6 are critical mediators in cellular proliferation, where they help to drive the transition of DNA synthetic phase of the cell-division cycle (8). Cyclin-dependent kinase 4/6 inhibitors (CDK4/6i) are a novel class of drugs targeting the dysregulated cell cycle in malignant cells, including Palbociclib, Ribociclib, and Abemaciclib. CDK4/6i are now commonly used as approved and investigative treatments across many cancer types. A previous study has demonstrated that Palbociclib treatment alters nucleotide biosynthesis and glutamine dependency in A549 cells (9). The previous study has shown that adenocarcinoma cell lines are more sensitive to CDK4 than squamous cancer cell lines (10).Unfortunately, recent clinical trials of CDK4/6i as single agents in non-small cell lung cancer (NSCLC) have not achieved satisfactory results (11–14). These disappointing results have prompted studies on the combinatorial strategy of CDK4/6i and other agents.

Pemetrexed is a cytostatic antifolate drug that inhibits thymidylate synthase (TS) and several other enzymes in the nucleotide synthesis pathway and is a cornerstone for the treatment of lung cancer (15). Ribociclib is one of the selective CDK4/6i that blocks tumor suppressor retinoblastoma protein (RB) phosphorylation and induces cell cycle arrest (16). Interestingly, a clinical evaluation of the combination therapy of abemaciclib and pemetrexed in a phase Ib trial has demonstrated an acceptable safety profile (17). However, whether the combination of ribociclib and pemetrexed has the same safety and enhanced anti-tumor effect needs further experimental confirmation.

Therefore, this study aimed to evaluate the efficacy and mechanism of action of ribociclib in combination with pemetrexed in LUAD cells. We found that ribociclib plus pemetrexed showed robust cytotoxicity and antitumor effect. Several molecular pathways that appeared to drive the combinatorial antitumor effect cumulatively were identified. Our findings supported clinical testing of this combination therapeutic strategy for lung adenocarcinoma patients.

## Materials and Methods

### Data Sources

The UALCAN (http://ualcan.path.uab.edu/) is a web-based tool for gene expression analysis of cancer OMICS data (18). The RNA sequencing (RNA-seq) information and corresponding clinical data of LUAD patients were downloaded from The Cancer Genome Atlas (TCGA) database (https://www.cancer.gov/tcga). Baseline clinicopathological information and final clinical outcome were recorded for each patient.

### Cell Lines and Reagents

The human lung adenocarcinoma cell lines A549, HCC827, NCI-1395, and NCI-H1650 were obtained from the American Type Culture Collection (ATCC). PC9 and NCI-H1975 cells were provided by Dr Fan (Department of Thoracic Surgery, Fourth Military Medical University, Xi’an, China). All cells were grown in Roswell Park Memorial Institute (RPMI) 1640 complete medium (Gibco, USA) supplemented with 10% fetal bovine serum (FBS; Gibco, USA), 100 U/mL penicillin (Thermo Fisher Scientific, MA, USA), and 100 ug/mL streptomycin (Thermo Fisher Scientific, MA, USA). All cells were cultured in a humidified atmosphere with 5% CO_2_ at 37°C. Cell lines were routinely authenticated, and mycoplasma tested. Ribociclib was purchased from Selleck, China, and pemetrexed was from Med Chem Express, China. Drugs were dissolved in dimethyl sulfoxide (DMSO, Sigma, USA) and stored at -20°C.

### Cell Viability Assay

A549 and PC9 cells were cultivated in RPMI 1640 complete medium. Approximately 2000 cells per well were seeded in 96-well plates. After overnight incubation, cells were exposed to a 10-fold serial dilution of ribociclib ranging from 0.01 to 100 μmol/L and pemetrexed ranging from 0.001 to 10 μmol/L for 6, 24, 48, and 72 h. Cell viability was detected using Cell Counting Kit 8 (CCK-8, Beyotime, China) following the manufacturer’s protocol. Cell viability was determined by the percentage of surviving drug-treated cells versus DMSO-treated control cells. The IC50 value was the concentration for 50% of maximal inhibition of cell proliferation. For two drugs combination experiments, cells were treated with indicated doses of ribociclib and/or pemetrexed (1:100) for 72h.

### Colony Formation Assay

For colony formation detection, cells were cultured and treated with 0.1 μmol/L pemetrexed and/or 10 μmol/L ribociclib for 72 h. Equal amounts of the solvent (DMSO) were added as a control group. Approximately 1000 single cells per well were seeded in 6-well plates and incubated for 12 days. At the endpoints of the colony formation experiment, the cells were washed twice with PBS, fixed with 4% paraformaldehyde (PFA, Sigma, USA) for 15 min, and stained using 0.2% crystal violet (Sigma, USA) for observation. All relevant assays were independently performed at least three times.

### Wound Healing Assay

A density of 1 × 10^5^ cells/mL PC9 or A549 cells were seeded in 6-well plates. After 24h incubation, the cells were either untreated or treated with 10μmol/L ribociclib and/or 0.1μmol/L pemetrexed. When the cell reached about 90–100% confluence, a sterile 200 μL pipette tip was used to scratch a line. The cells were washed twice with PBS and cultured in a fresh medium with 0.1% FBS. Wound healing was observed at 0h and 48 h using a microscope (Olympus, Japan) and analyzed using Image J 1.8.0.

### Transwell Migration and Invasion Assay

Cell invasion assays were performed using 24-well transwells (8μm pore size; Corning, USA) and coated with 50μL diluted Matrigel (1:5 in PBS) (BD Biosciences, USA). Cells were either untreated or treated with 10μmol/L ribociclib and/or 0.1μmol/L pemetrexed for 72h. Then, the upper chamber added A549 and PC9 cells (5 × 10^4^ cells/200μL cell suspension in FBS-free medium). The lower chambers filled 500 μL of RPMI 1640 medium containing 10% FBS. After 24h incubation, the cells in the upper chamber were removed with cotton swabs, and the cells adherent to the bottom surface were fixed with cold 4% PFA for 15 min and stained using 0.2% crystal violet. Finally, after washing the filters in water, five random fields/filters were taken and counted under a microscope (Olympus, Japan) with a 100-fold magnification.

### Cell Cycle Analysis

Approximately 1 × 10^5^ cells per well Cells were seeded into 6-well plates. After 24h incubation, cells were either untreated or treated with 10μmol/L ribociclib and/or 0.1μmol/L pemetrexed for 72h. Then adherent cells were harvested, washed, and resuspended in cold PBS. Single-cell suspensions were overnight fixed in cold 70% ethanol at 4°C. The fixed cells were washed with PBS and stained with 50 μg/mL propidium iodide (Beyotime, China) containing 50 μg/mL RNase I for 30 min at room temperature and then analyzed using flow cytometry (BD AriaIII, USA) and Flowjo software.

### Cell Apoptosis Analysis

According to the manufacturer’s instructions(C1062s, Beyotime, China), The apoptotic status of A549 and PC9 cells was tested using flow cytometry *via* the Annexin V-fluorescein isothiocyanate (FITC) and PI double staining method. Briefly, the cells were seeded into six-well plates at a density of approximately 1 × 10^5^ cells per well and treated with 0.1μM pemetrexed and/or 10 μM ribociclib for 72 h. The cells were then collected and resuspended in 500 μL of binding buffer containing 5 μL Annexin V-FITC and 5 μL PI, and then incubated for 15–30 min in the dark at room temperature and analyzed using flow cytometry.

### RNA Isolation and qRT-PCR

Total RNA from cells was extracted with Total RNA Kit II (R6934-01, Omega, Georgia, USA). RNA samples were quantified with Nanodrop. cDNAs were synthesized with a Reverse Transcription Kit (RR036A, Takara Biotechnology, Japan),. Quantification of gene expression was performed with SYBR Premix Ex Taq II (RR820A, Takara Biotechnology, Japan). Gene expression levels were quantified using the delta-delta CT method with GAPDH as a housekeeping gene. The primers used to amplify the indicated genes are listed in Supplementary Material (**Supplementary Table 1**).

### Western Blotting Analysis

Western blotting was performed as previously described (19). Cells were treated with the drug corresponding to the drug treatment group, DMSO control, 10 μM ribociclib, 0.1μM pemetrexed, 10 μM ribociclib +0.1μM pemetrexed. After 72 hours, cells were harvested and lysed in RIPA buffer (Beyotime, China) in the presence of protease/phosphatase inhibitor (Roche, USA). Protein concentration was quantified using a BCA assay (Thermo Fisher Scientific, Italy). The lysates were mixed with 1x loading buffer (Beyotime, China). A total of 30 μg protein per sample was loaded to 7.5% and 10% SDS-PAGE, which was run for one hour at 150V in the running buffer (25 mM Tris base, 192 mM glycine, 0.1% SDS). Then, the protein from gels was transferred to an activate PVDF membrane (Tanon, Shanghai, China) running for 60 min at 100V in the transfer buffer (25 mM Tris base,192 mM glycine, 10% methanol). Membranes were blocked with 5% BSA for 1h. Subsequently, protein was overnight incubated at 4°C with the following primary antibodies: Antibodies against CDK4 (CST-23972,1:1000 dilution), CDK6 (CST-13331T,1:1000), phospho-Rb ser807/811 (CST-8516T, 1:1000), Cyclin D1 (CST-55506T, 1:1000), E2F1 (CST-3742S, 1:1000), Cleaved Caspase-3 (CST-9664T, 1:1000), Cleaved Caspase-9 (CST-9505T, 1:1000), Cleaved PARP-1 (CST-5625T, 1:1000), Bcl-2 (CST-4223S, 1:1000), Vimentin (CST-5741S, 1:1000), E-cadherin (CST-3195S, 1:1000), β-actin (CST-3700S,1:1000) were purchased from Cell Signal Technology (Danvers, MA, USA). Membranes were incubated with HRP coupled goat anti-mouse or goat anti-rabbit secondary antibody (1:2000 dilution, Proteintech, Wuhan, China) for 60 min at room temperature. The immunoreactive proteins were detected using enhanced chemiluminescence (P0018S, Beyotime, China).

### Immunofluorescence (IF) Staining

For immunofluorescence staining, A549 and PC9 cells were seeded on 10 mm confocal dishes and treated with 0.1 μmol/L pemetrexed and/or 10 μmol/L ribociclib. Equal amounts of DMSO were added to control cells. After 72h, cells were fixed in 4% PFA for 30 min, permeabilized in 0.3% Triton X-100 for 20 min, and blocked in 5% normal goat serum for 60 min at room temperature. Sequentially, cells were incubated with primary antibody against CDK4 (1:200 dilution, #ab108357, Abcam, USA) or phosphor-Rb (1:200, #8516T, Cell signalling Technology, USA) at 4°C overnight, then washed with phosphate buffered-saline with Tween-20 (PBST) and incubated with FITC-labelled secondary antibody (1:100 dilution, Proteintech, Wuhan, China) for 1 h at room temperature. The nuclei were labelled using 4′,6-diamidino-2-phenylindole (DAPI) (2 mg/mL) in the dark for 15 min, and imaging was performed on a fluorescence microscope (Olympus IX 73 DP80, Japan).

### Immunohistochemistry (IHC)

Immunohistochemical analyses were performed on specimens from xenograft tumors. Formalin-fixed paraffin-embedded tissue sections (4μm thickness) were deparaffinized and rehydrated in graded ethanol solutions (100%, 95%, 80%, 70%). For IHC staining, after antigen repair, the tissue slides were incubated with anti-Ki-67 (1:200, #9027S, Cell signaling, USA) overnight at 4°C, then washed with PBS and incubated with a horseradish peroxidase-labelled secondary antibody for 30 min and stained with diaminobenzidine (DAB) for 5 min. For H&E staining, tissue sections were stained with hematoxylin for 3 min and eosin solution for 30 s. For the TdT-mediated dUTP nick-end labelling (TUNEL) assay, we used a TUNEL *in situ* apoptosis detection kit (Roche, USA) according to the manufacturer’s instructions. The stained images were observed using a fluorescence microscope at a 200-fold magnification.

### Animal Experiments

Female BALB/C nu/nu mice (6-week-old) were purchased from Vital River Laboratory Animal Technology (Beijing, China), and all animal experiments were approved by the Fourth Military Medical University Animal Care Facility and were performed according to National Institutes of Health guidelines. For the synergic effect test of ribociclib combined with pemetrexed, the method performed is as follows. A total of 5 × 0^6^ A549-luciferase cells were collected in 100 μL PBS and subcutaneously injected into the right flank of each mouse. When the tumor size reached about 100 mm^3^, the mice were randomized into 4 groups (n=5 per group). they were treated as described in the following: The first group of mice were intratumorally injected with saline solution as an untreated vehicle (Control), the second was treated with ribociclib (200 mg/kg, oral, 21 d) alone (Ribo), the third received pemetrexed (100 mg/kg) administered alone (PTX), the fourth received ribociclib and pemetrexed combination treatment (Ribo + PTX) and the size of the subcutaneous tumors and weight of the mice were recorded every 4 days. Tumor volume (V) was calculated according to the formula: Π/6 × length × width^2^.

### Statistical Analysis

The expression of CDK4 was recorded as a dichotomous (high vs low) variable by the optimal cut-off value using Z-score. Survival curves according to CDK4 expression were estimated with the Kaplan-Meier method and a log-rank test was used to assess significance. All statistical analyses were performed using SPSS Statistics 21. Unless stated otherwise, all experiments were conducted in triplicate. Data were expressed as the mean ± standard deviation (SD) of at least three independent experiments. The significance of differences between mean values was determined using a two-way analysis of variance (ANOVA) with Tukey’s *post hoc* multiple comparisons, depending on the normality of data distribution. Statistical significance was set at a P value less than 0.05.

## Results

### CDK4 Expression Is Associated With Poor Prognosis in LUAD

**Figure 1:**
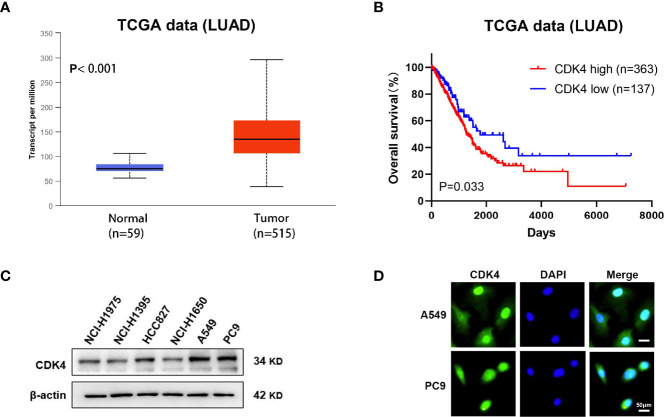
Elevated expression of CDK4 indicates a poor prognosis in patients with LUAD. **(A)** Representative data extracted from TCGA datasets showing the relative expression of CDK4 mRNA in LUAD tissues compared with normal tissues. **(B)** Kaplan–Meier analysis showing the correlation between CDK4 mRNA expression and OS for the patients with LUAD included in TCGA datasets. **(C)** Western blotting analysis was performed using different LUAD cells. **(D)** IF staining for CDK4 in A549 and PC9 cell lines. The scale bars represent 50 μm. LUAD, lung adenocarcinoma; TCGA, The Cancer Genome Atlas; OS, overall survival; IF, immunofluorescence.

We first determined the gene expression levels of CDK4 using The Cancer Genome Atlas (TCGA) datasets. CDK4 mRNA expression levels in LUAD were significantly higher than in normal control tissues (p < 0.001, **Figure 1A**). Furthermore, Kaplan–Meier curves analysis of TCGA samples showed that higher CDK4 mRNA expression levels were correlated with poorer overall survival (OS) (p=0.033, **Figure 1B**), strongly suggesting that CDK4 contributes to LUAD progression. Subsequently, we detected the protein expression of CDK4 in a panel of 6 LUAD cells (NCI-H1975, NCI-H1395, HCC827 NCI-U1650, A549 and PC9), and we found that CDK4 was highly expressed in A549 and PC9 cell lines (**Figure 1C**) and thus, A549 and PC9 cell lines were chosen for further studies. Immunofluorescence assay was conducted to assess the subcellular location of CDK4.The result showed that CDK4 was primarily localized in the nucleus with a low expression in the cytoplasm in A549 and PC9 cells. We also detected the protein expression of CDK4 in BEAS-2B, a normal human lung epithelial cell line, and found that CDK4 was low expressed in BEAS-2B (**Figure 1D** and **Supplementary Figure 1**). Taken together, these findings suggest that CDK4 expression is upregulated in LUAD and indicates a poor prognosis. A549 and PC9 cells can be used as suitable cell models for this study.

### Ribociclib Combined With Pemetrexed Shows Enhanced Cytotoxicity in LUAD Cells

**Figure 2:**
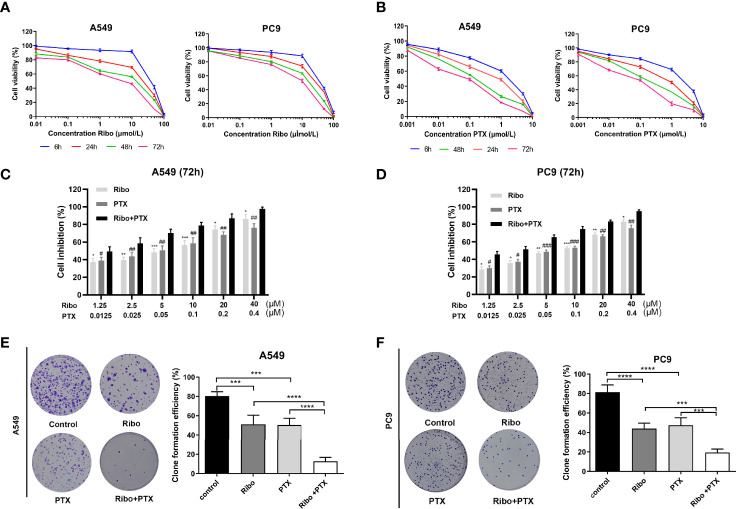
Ribociclib plus pemetrexed enhanced the cytotoxicity *in vitro.* **(A, B)** A549 and PC9 cell lines were exposed to ribociclib and pemetrexed in combination or alone at different doses. **(C, D)** Cell lines were exposed to different combinations of pemetrexed and ribociclib dosages (*p < 0.05, **p < 0.01, and ***p < 0.001, ****p < 0.0001), ribociclib (Ribo) versus Ribo + pemetrexed (PTX) (#p < 0.05, ##p < 0.01, and ###p < 0.001), and PTX versus Ribo + PTX). **(E, F)** Representative images and quantification of colony formation assay. Data are presented as mean ± SD of triplicate experiments (*p < 0.05, **p < 0.01, ***p < 0.001 as ***p < 0.001,****p < 0.0001). SD: standard deviation.

In our study, the antiproliferative effects of using A549 and PC9 cells of the Ribo and PTX alone groups were concentration- and time-dependent (**Figures 2A, B**). The IC50 values of A549 and PC9 cell lines of the Ribo group were 45.56 ± 0.811 μM (6 h), 15.98 ± 0.1466 μM (24 h), 4.796 ± 0.0637 μM (48 h), and 2.104 ± 0.0539 μM (72 h) and 38.6 ± 0.194 μM (6 h), 23.37 ± 0.1467 μM (24 h), 12.34 ± 0.1144 μM (48 h), and 6.165 ± 0.067 μM (72 h), respectively. The IC50 values of A549 and PC9 cell lines of the PTX group were 1.064 ± 0.07388 μM (6 h), 0.3864 ± 0.0487 μM (24 h), 0.1245 ± 0.02606 μM (48 h), and 0.0499 ± 0.029 μM (72 h) and 1.968 ± 0.134 μM (6 h), 0.5445 ± 0.0543 μM (24 h), 0.206 ± 0.0311 μM (48 h), and 0.0766 ± 0.0315 μM (72 h), respectively. We then evaluated the growth inhibition effect of Ribo + PTX group at different concentrations for 72 h. The results showed that the growth inhibition effect of Ribo + PTX group was more potent than that of Ribo or PTX group at each concentration (**Figures 2C, D**). Colony formation is an important parameter in cancer survival and development, and thus, we next evaluated these effects by conducting colony formation assays. A549 and PC9 cells of Ribo + PTX group inhibited colony formation to a greater extent than Ribo or PTX group (**Figures 2E, F**). These findings suggested that ribociclib combined with pemetrexed shows enhanced cytotoxicity in LUAD cells.

### Ribociclib and Pemetrexed Combination Enhances LUAD Cell Death *Via* the Caspase/Bcl-2 Signalling Pathway

**Figure 3:**
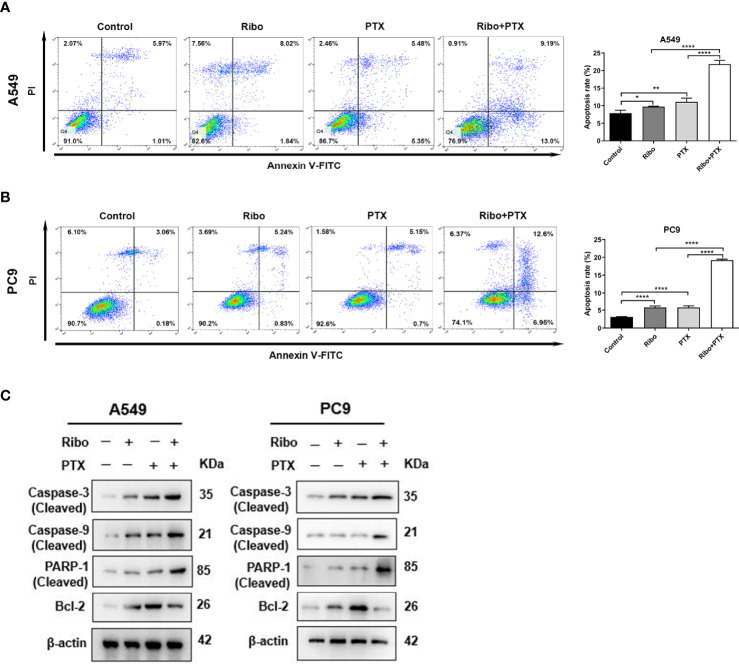
The effect of ribociclib plus pemetrexed on inducing apoptosis of A549 and PC9 cells *in vitro.* **(A, B)** A549 and PC9 cells were treated with ribociclib with or without pemetrexed. The quantitative analysis was shown in the bar graphs. **(C)** The levels of the apoptosis-related proteins were analyzed using western blotting in A549 and PC9 cells treated with or without ribociclib with or without pemetrexed. Data are presented as mean ± SD of triplicate experiments (*p < 0.05, **p < 0.01, ****p < 0.0001). SD, standard deviation.

Apoptosis is one of the main mechanisms of cancer cell death. We next decided to examine the effect of ribociclib in combination with pemetrexed on apoptosis. A549 and PC9 cells were treated with 10 μM Ribo and 0.1 μM PTX together or alone for 72 h. Our results showed that a high percentage of cells undergoing apoptosis was observed in the Ribo + PTX group (**Figures 3A, B**). To elucidate the potential molecular mechanisms, we next analyzed the mRNA and protein levels of the caspase family and Bcl-2, which are involved in apoptosis. Our results found that the expression of cleaved caspase 3, cleaved caspase 9, and cleaved PARP were upregulated, whereas the expression of bcl-2 was downregulated, and the changes in the levels of these proteins and mRNA were more obvious in the cells of the Ribo + PTX group than treatment with Ribo or PTX alone (**Figure 3C** and **Supplementary Figure 3**). The results revealed that ribociclib combined with pemetrexed enhances cell death *via* the Caspase/Bcl-2 signalling pathway.

### Ribociclib and Pemetrexed Coadministration Leads to Cell-Cycle Arrest

**Figure 4:**
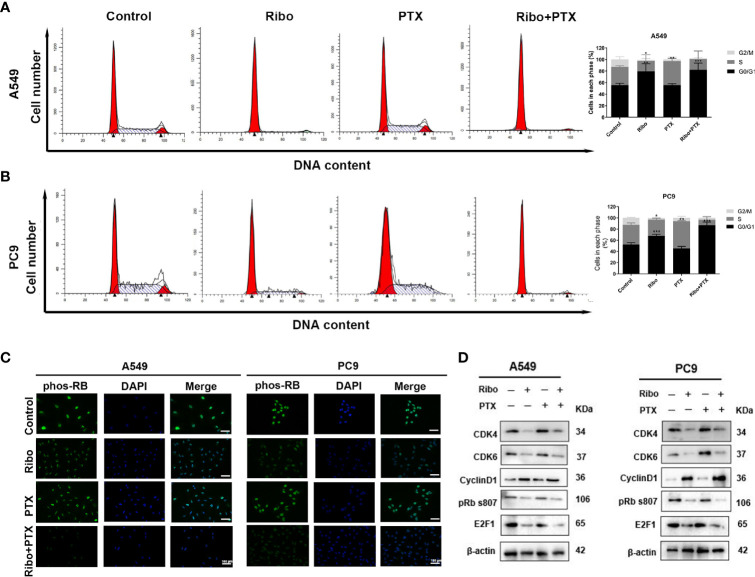
The effect of ribociclib and pemetrexed on cell cycle in A549 and PC9 cells. **(A, B)** The cell cycle distribution of A549 and PC9 cells treated with ribociclib and pemetrexed alone or in combination. **(C)** The expression and subcellular localization of pRB were detected using immunofluorescence analysis in A549 and PC9 cells. **(D)** Western blotting showed the changes in levels of cyclin D1, CDK4/6, pRB, and E2F1. The scale bars represent 100 μm. Data are presented as mean ± SD of triplicate experiments (*p < 0.05, **p < 0.01, and ***p < 0.001). SD, standard deviation.

The cell cycle distribution was analyzed in cells of the Ribo, PTX, and Ribo + PTX groups by flow cytometric and western blot analysis. The Ribo group experienced a G1-phase arrest in ~69% of A549 cells and ~65% of PC9 cells. The PTX group experienced an S-phase arrest in ~46% of A549 cells and ~49% of PC9 cells. The Ribo + PTX group experienced a G1-phase arrest in ~95% of A549 cells and ~91% of PC9 cells (**Figures 4A, B**). To further identify the regulatory mechanism and confirm the effect of the drugs on cell cycle distribution, we first conducted an immunofluorescence analysis to visualize the expression and subcellular localization of phosphorylated retinoblastoma (Phos-RB) protein, a vital molecule of the CDK4/Cyclin D/RB/E2F pathway. **Figure 4C** shows that Phos-RB mainly was localized in the nucleus, and treatment with the Ribo and PTX significantly inhibited the phosphorylation of RB. Furthermore, we detected the expression of various cell cycle-related proteins and mRNA levels. We found that the expression levels of CDK4, CDK6, phos-RB, and E2F1 were downregulated and that of cyclinD1 was upregulated, consistent with G1-phase arrest (**Figure 4D** and **Supplementary Figure 3**). These results suggest that concurrent administration of Ribo and PTX results in cell cycle distribution through the CDK4/Cyclin D/RB/E2F pathway.

### Ribociclib Combined With Pemetrexed Inhibits Cell Migration and Invasion

**Figure 5:**
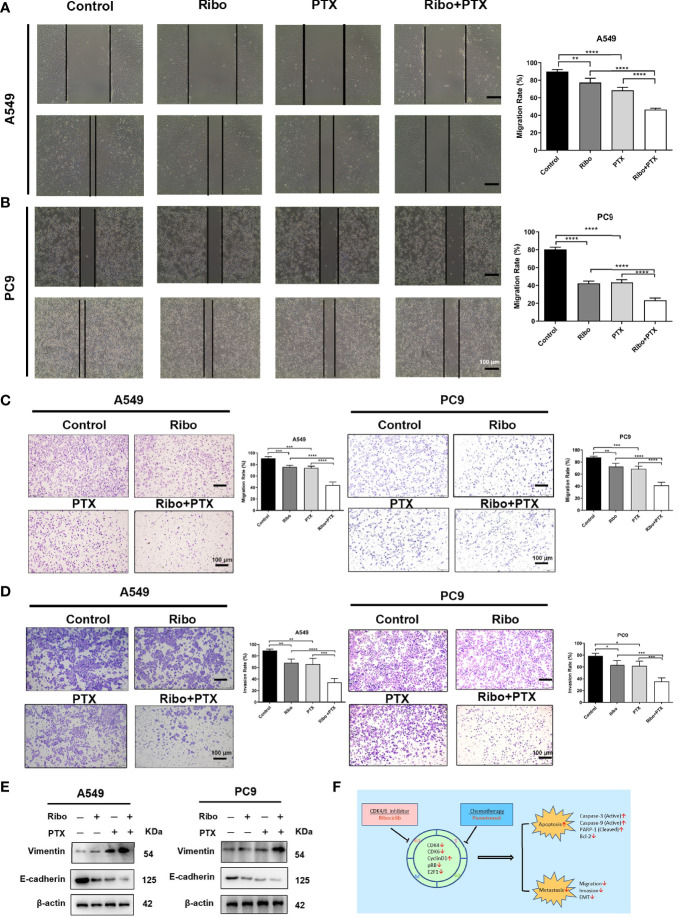
The inhibition effect of ribociclib plus pemetrexed on the migration and invasion of A549 and PC9 cells. **(A, B)** Representative images and quantification of wound healing assay results using A549 and PC9 cells. **(C, D)** Representative images and quantification of transwell migration and invasion assays results using A549 and PC9 cells. **(E)** The EMT-related protein molecules were analyzed using western blotting in A549 and PC9 cells. The scale bars represent 100 μm. Data are presented as mean ± SD of triplicate experiments (*p < 0.05, **p < 0.01, ***p < 0.001, and ****p < 0.0001). **(F)** Summary of the effects of treatments with ribociclib and pemetrexed on their molecular targets and the different observed outcomes. EMT, epithelial to mesenchymal transition; SD, standard deviation.

Effects of Ribo and PTX on cell migration were detected using wound healing and transwell assays. Our results showed that A549 and PC9 cells treated with combination with Ribo plus PTX were significantly reduced cell migration compared with those of the Ribo group or PTX group (**Figures 5A−C**). The results of transwell invasion assay showed that the cell migration of the Ribo + PTX group was significantly lower than that of Ribo or PTX groups (**Figure 5D**). As epithelial to mesenchymal transition (EMT) is a key process for metastasis, we next examined the expression of EMT-related proteins (E-cadherin and vimentin) using western blotting. Our results showed that vimentin expression was downregulated whereas E-cadherin was upregulated in cells treated with the Ribo or PTX alone, which were statistically significant than those in the Ribo + PTX group (**Figures 5E, F**). These data suggested that ribociclib combined with pemetrexed significantly inhibits cell metastasis.

### Ribociclib Combined With Pemetrexed Promotes Antitumor Effect in a Xenograft Mouse Model

**Figure 6:**
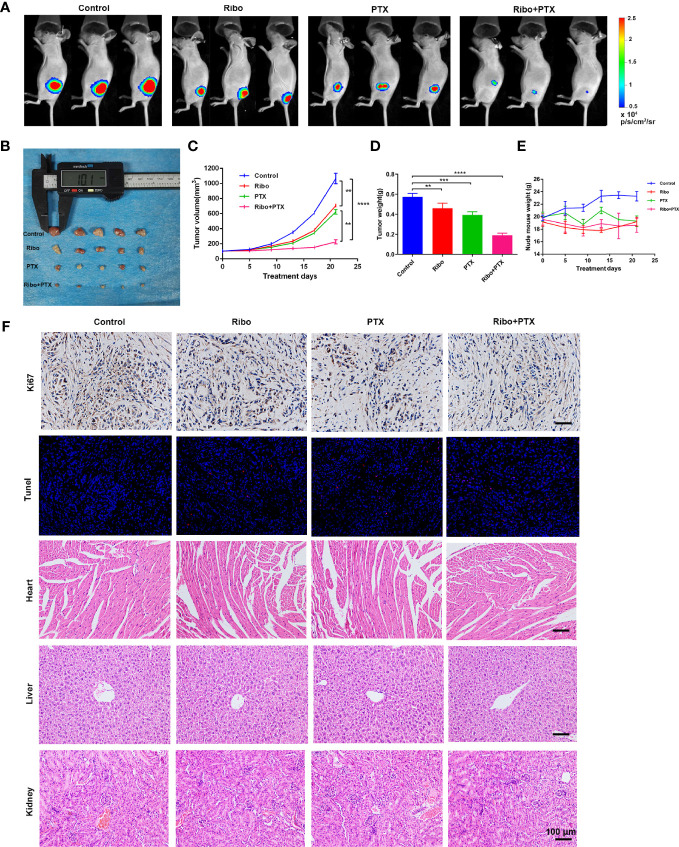
The therapeutic effect of ribociclib plus pemetrexed in a xenograft nude mouse model. **(A)** Representative fluorescence images of luciferase signals captured from subcutaneous tumors are shown. **(B–D)** The change of tumor volume and tumor weights (n = 5 mice per group). **(E)** The effect of body weight. Data are presented as mean ± SD of triplicate experiment (**p < 0.01, ***p < 0.001 and ****p < 0.0001). **(F)** Representative panels of immunohistochemical and HE staining. The scale bars represent 100 μm. SD, standard deviation; HE, hematoxylin and eosin.

Based on the results from *in vitro* studies, we further evaluated whether combined ribociclib and pemetrexed treatment could enhance the anti-tumor effect in LUAD xenograft mouse models. The tumor volume and tumor weight were decreased in groups treated with Ribo or PTX alone compared to the control group, and further decreased in the Ribo + PTX group (**Figures 6A−D**). In addition, no appreciable detrimental effects or abnormal symptoms were observed during the drug treatments based on the body weight (**Figure 6E**). Moreover, damage to the vital organs such as heart, liver, and kidney was not observed using hematoxylin and eosin (HE) staining (**Figure 6F**). We next analyzed the expression of Ki67 (a marker for cell proliferation) in the tumor sections by IHC. Although treatment by ribociclib or pemetrexed alone downregulated the expression of Ki67, the combination therapy was more effective (**Figure 6F**). The TdT-mediated dUTP-biotin nick end labelling (TUNEL) analyses revealed that coadministration of ribociclib and pemetrexed effectively increases apoptosis in xenograft tumors (**Figure 6F**). Taken together, the combination of ribociclib and pemetrexed yielded a superior response in the xenograft LUAD model.

## Discussion

In this study, we evaluated the response of LUAD cells, A549 and PC9, to ribociclib and pemetrexed. We found that the combination treatment of ribociclib and pemetrexed showed an enhanced effect on cell proliferation, cell cycle distribution, cell migration, cell invasion, and cell death. Our results suggested that the combination of ribociclib and pemetrexed led to a synergistic effect. In addition, we found that the anti-tumor effect is mediated through modulation of the CDK4/Cyclin D/RB/E2F and Caspase/Bcl-2 signal pathways.

Treatment options for patients with metastatic NSCLC on or after first-line treatment are limited considerably after cancer progression. Among the available treatments, historical median progression-free survival is only 2.0–4.5 months for second-line treatment and likely shorter for the subsequent treatment (20), which makes the treatment of these patients challenging.

The cell cycle dysregulation frequently occurs in lung cancers (7, 21). CDKs are critical cell cycle regulators and drive cellular proliferation through the most complex molecular interactions (22). Aberrant activation of CDKs provided a rationale for CDKs inhibitors to be used as anticancer drugs in advanced NSCLC. Recently, the development of CDK4/6i (palbociclib, ribociclib, and abemaciclib) and the approval of their use by the Food and Drug Administration (FDA) for advanced metastatic breast cancer have resulted in the designing of multiple clinical trials using these agents (23). Many clinical trials of CDK4/6i in several tumor types have achieved promising results (24–26). Unfortunately, CDK4/6i in human NSCLC have demonstrated little clinical activity (11, 12). Due to the unsatisfactory results of single agents for NSCLC, the combination of CDK4/6i and other conventional therapy is being tested in clinical trials to enhance anti-tumor efficacy. A recent study identifies that palbociclib could sensitize lung cancer cells to EGFR-TKI and gefitinib (27). Co-treatment with MEK inhibitor (trametinib) plus palbociclib has shown significant anti-KRAS-mutant and anti-CDKN2A-mutant NSCLC activities in preclinical models (28). In addition, as the critical role in cell proliferation and progression, mTOR inhibitors are considered promising candidates for synergistic inhibitory effects with CDK4/6i (29). Currently, abemaciclib combined with pemetrexed has demonstrated an acceptable safety profile in a clinical evaluation in a phase Ib trial (17). However, practical strategies for formulating rational trial designs have not been identified. Thus, well-planned experiments using suitable animal and cell models are still needed.

CDK4 gene amplification has been found in numerous types of cancer including breast cancer (30), sarcoma (31), cervical cancer (32), and melanoma (33). The effect of CDK4 amplification on CDK4/6i sensitivity remains controversial; although it enhanced sensitivity in liposarcoma (34), it caused resistance in glioblastoma (35). This study found that CDK4 was highly expressed in LUAD cells and is associated with poor patient outcomes in the TCGA dataset. In theory, CDK4/6i could be combined with cytotoxic agents that target the S or M phase of the cell cycle to kill tumor cells. However, recent studies indicated that CDK4/6i and chemotherapeutic drugs might have antagonistic effects. Exposure of RB-intact breast cancer cells to palbociclib prior to cytotoxic agents (doxorubicin or carboplatin) significantly reduced their cytotoxicity (36). Another study demonstrated that the combination of palbociclib and taxanes at clinically available doses in multiple squamous cell lung cancer models enhanced antitumor effects by inhibiting the pRB-E2F signalling pathway (5). Studies of combination therapy of CDK4/6i with cytotoxic chemotherapy using pemetrexed have not been reported. A549 and PC9 cells are typical lung adenocarcinoma cells and express wild-type RB, which harbour a p16^INK4A^ deletion resulting in constitutive RB hyperphosphorylation and inactivation. Our study found that ribociclib combined with pemetrexed shows enhanced cytotoxicity in A549 and PC9 cells. Combining these two classes of drugs did not demonstrate antagonistic effects. Pemetrexed blocked cells in the S phase, whereas ribociclib arrested cells in the G1 phase. Concomitant treatment showed more robust G1 phase arrest and pro-apoptosis effects than treatment with ribociclib or pemetrexed alone. We speculate that the CDK4/6-cyclinD-pRB-E2F pathway regulated the cell cycle; however, more evidence is needed to confirm the same. In addition, we also found that treatment with ribociclib and pemetrexed in combination showed significant inhibitory effects on the migration and invasion of A549 and PC9 cells. In the LUAD xenograft mouse model, the coadministration of ribociclib and pemetrexed amplified the anti-tumor effect without increasing toxicity. Taken together, Our results showed that ribociclib combined with pemetrexed had strong cytotoxicity, antitumor effect and acceptable safety, indicating the potential practicability of combination therapy.

Clinical trials evaluating combinations of CDK4/6 inhibitors with other agents have accumulated in recent years (37). However, the effects of CDK4/6 inhibition are far more wide-reaching. New insights into their mechanisms of action have triggered the identification of new therapeutic opportunities, including the development of new combinations and modification of dosing schedules (38). This will extend the utility of CDK4/6 inhibitor to the treatment of other cancer types.

## Conclusions

Our studies demonstrate that the pharmacologic inhibition of CDK4/6 by ribociclib in combination with pemetrexed leads to improved therapeutic responses. The combinatorial effect of these drugs may be through the modulation of the CDK4/6-cyclinD-pRB-E2F pathway. Although the relevant proteins have undergone significant changes, whether these factors may play a role in ribociclib plus pemetrexed combination treatment warrants further investigation. Currently, only the tolerance and benefit of abemaciclib plus pemetrexed have been investigated clinically. A positive outcome of this study will provide further support for the assessment of ribociclib and pemetrexed combination in LUAD. Meanwhile, this study also supplies potential treatment options for patients with advanced LUAD.

## Data Availability Statement

The original contributions presented in the study are included in the article/**Supplementary Material**. Further inquiries can be directed to the corresponding authors.

## Ethics Statement

The animal study was reviewed and approved by The Ethics Committee of Air Force Military Medical University.

## Author Contributions

Conception and design, YK, C-GL, and Z-QZ. Administrative support, L-MK and H-LZ. Collection and assembly of data, YK and R-JL. Data analysis and interpretation, X-ML and H-LZ. All authors contributed to the article and approved the submitted version.

## Funding

This study was supported by grants from the Basic Research Plan of Natural Science in Shaanxi Province(2020JM-326), the National Natural Science Foundation of China (8217111294) and the Social Talent Fund Supporting Scheme of Tangdu Hospital (2021SHRC001), the Key Research and Development Plan of Natural Science in Shaanxi Province(2020SF-066).

## Conflict of Interest

The authors declare that the research was conducted in the absence of any commercial or financial relationships that could be construed as a potential conflict of interest.

## Publisher’s Note

All claims expressed in this article are solely those of the authors and do not necessarily represent those of their affiliated organizations, or those of the publisher, the editors and the reviewers. Any product that may be evaluated in this article, or claim that may be made by its manufacturer, is not guaranteed or endorsed by the publisher.

## Abbreviations

CDK4/6, cyclin-dependent kinase 4/6; NSCLC, non-small cell lung cancer; LUAD, lung adenocarcinoma; TCGA, The Cancer Genome Atlas; EGFR, epidermal growth factor receptor; BRAF, v-raf murine sarcoma viral oncogene homolog B1; ALK, anaplastic lymphoma tyrosine kinase; ROS-1, proto-oncogene tyrosine protein kinase 1; RB, retinoblastoma; Ribo, ribociclib; PTX, pemetrexed; PFS, progression free survival; MEK, mitogen activated protein kinase; mTOR, mammalian target of rapamycin.
