# Supplementary material for: Combining a CDK4/6 Inhibitor With Pemetrexed Inhibits Cell Proliferation and Metastasis in Human Lung Adenocarcinoma
Source: Front Oncol. 2022 May 24;12:880153. doi: 10.3389/fonc.2022.880153 (PMC9172583; doi:10.3389/fonc.2022.880153)
Supplement: Supplementary file 1 [file DataSheet_1.docx]

**
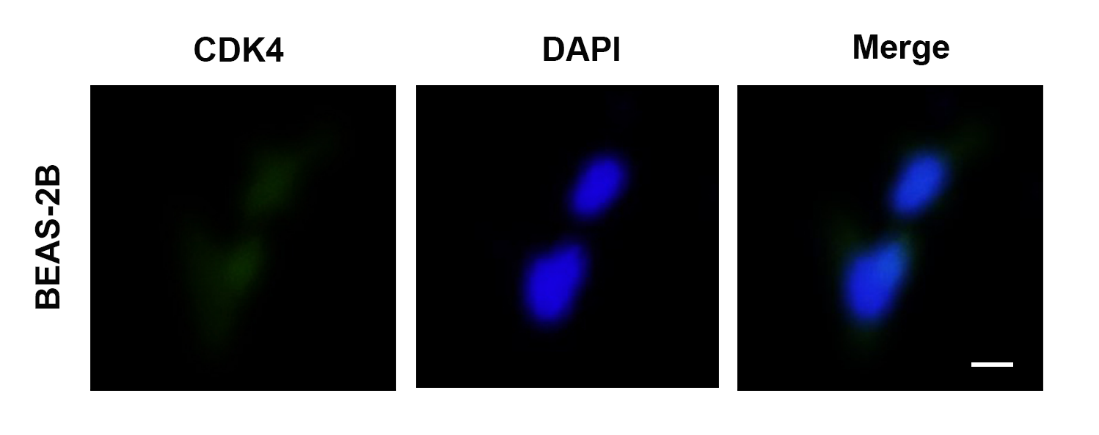
Supplementary Figure 1 (relate to Figure 1). CDK4 expression analysis in BEAS-2B cells.**

**

**

**Supplementary Figure 2（relate to Figure 3）. Relative mRNA expression analysis of Caspase-3, Caspase-9, Bcl-2, and Ki-67 gene in A549 and PC9 cells. A.** A549 cells were treated with 10 μM ribociclib (Ribo）and/or 0.1μM pemetrexed (PTX) for 72h, respectively (****p<0.001). **B.** PC9 cells were treated with 10 μM ribociclib (Ribo）and/or 0.1μM pemetrexed (PTX) for 72h, respectively (****p<0.001).

**

**

**Supplementary Figure 3 (relate to Figure 4). Relative mRNA expression analysis of CDK4, CDK6, Cyclin D1 gene in A549 and PC9 cells. A.** A549 cells were treated with 10 μM ribociclib (Ribo）and/or 0.1μM pemetrexed (PTX) for 72h, respectively (****p<0.001). **B.** PC9 cells were treated with 10 μM ribociclib (Ribo）and/or 0.1μM pemetrexed (PTX) for 72h, respectively (****p<0.001).
