# Supplementary material for: Combining a CDK4/6 Inhibitor With Pemetrexed Inhibits Cell Proliferation and Metastasis in Human Lung Adenocarcinoma
Source: Front Oncol. 2022 May 24;12:880153. doi: 10.3389/fonc.2022.880153 (PMC9172583; doi:10.3389/fonc.2022.880153)
Supplement: Supplementary file 2 [file Table_1.docx]

**Supplementary Table 1. Primer sequences for real-time PCR analysis**

| **Gene** | **Primer sequence (5’- 3’)** |
| --- | --- |
| CDK4 | F: GAGCATGTAGACCAGGACCTAAG  R: GTTCCACCACTTGTCACCAGAAT |
| CDK 6 | F: AGGTGGCCCTCGGAATAGAT  R: GCCTGTTCCCACTACTCCAC |
| Cyclin D1 | F: CCTCGGTGTCCTACTTCAAATGT  R: TTCATCTTAGAGGCCACGAACAT |
| Caspase-3 | F: TGAGCCATGGTGAAGAAGGAATAA  R: CCCGGGTAAGAATGTGCATAAAT |
| Caspase-9 | F: TGTCCTACTCTACTTTCCCAGGT  R: CCCTTTCACCGAAACAGCATTAG |
| BCL-2 | F: GGATTGTGGCCTTCTTTGAGTTC  R: CTTCAGAGACAGCCAGGAGAAAT |
| Ki-67 | F: AGGGAAAGGAGAAGCAGGAAATT  R: TGTCCTCAGCCTTCTTTGGATTT |
| GAPDH | F: GCACCACCAACTGCTTAGCA  R: GTCTTCTGGGTGGCAGTGATG |
